# Delivery Practices and Associated Factors among Mothers Seeking Child Welfare Services in Selected Health Facilities in Nyandarua South District, Kenya

**DOI:** 10.1186/1471-2458-11-360

**Published:** 2011-05-21

**Authors:** Carol Wanjira, Moses Mwangi, Evans Mathenge, Gabriel Mbugua, Zipporah Ng'ang'a

**Affiliations:** 1 Institute of Tropical Medicine and Infectious Diseases, Jomo Kenyatta University of Agriculture and Technology, P.O Box 62000-00200, Nairobi, Kenya; 2 Centre for Public Health Research, Kenya Medical Research Institute, P.O Box 20742 - 00200, Nairobi, Kenya; 3 Centre for Microbiology Research, Kenya Medical Research Institute, P.O Box 54840 - 00200, Nairobi, Kenya; 4 Division for Malaria Control, Ministry of Health, P.O. Box 20750, Nairobi, Kenya

## Abstract

**Background:**

A measure of the proportion of deliveries assisted by skilled attendants is one of the indicators of progress towards achieving Millennium Development Goal (MDG) 5, which aims at improving maternal health. This study aimed at establishing delivery practices and associated factors among mothers seeking child welfare services at selected health facilities in Nyandarua South district, Kenya to determine whether mothers were receiving appropriate delivery care.

**Methods:**

A hospital-based cross-sectional survey among women who had recently delivered while in the study area was carried out between August and October 2009. Binary Logistic regression was used to identify factors that predicted mothers' delivery practice.

**Results:**

Among the 409 mothers who participated in the study, 1170 deliveries were reported. Of all the deliveries reported, 51.8% were attended by unskilled birth attendants. Among the deliveries attended by unskilled birth attendants, 38.6% (452/1170) were by neighbors and/or relatives. Traditional Birth Attendants attended 1.5% (17/1170) of the deliveries while in 11.7% (137/1170) of the deliveries were self administered. Mothers who had unskilled birth attendance were more likely to have <3 years of education (Adjusted Odds ratio [AOR] 19.2, 95% confidence interval [CI] 1.7 - 212.8) and with more than three deliveries in a life time (AOR 3.8, 95% CI 2.3 - 6.4). Mothers with perceived similarity in delivery attendance among skilled and unskilled delivery attendants were associated with unsafe delivery practice (AOR 1.9, 95% CI 1.1 - 3.4). Mother's with lower knowledge score on safe delivery (%) were more likely to have unskilled delivery attendance (AOR 36.5, 95% CI 4.3 - 309.3).

**Conclusion:**

Among the mothers interviewed, utilization of skilled delivery attendance services was still low with a high number of deliveries being attended by unqualified lay persons. There is need to implement cost effective and sustainable measures to improve the quality of maternal health services with an aim of promoting safe delivery and hence reducing maternal mortality.

## Background

Each year, approximately 536,000 women die from complications related to pregnancy and childbirth, with 99% of these deaths occurring in Africa and Asia. Slightly more than half of these deaths (270 000) occur in sub-Saharan Africa [1]. To make the achievement of the fifth Millennium Development Goal (MDG) a reality, Maternal Mortality Rates (MMR) will have to decrease at a much faster rate especially in sub-Saharan Africa, where the annual decline has so far been about 0.1% [2]. The recently released 2008/9 Kenya Demographic and Health Survey (KDHS) reported MMR of 488/100,000 which is an increase from the 2003 survey which reported an MMR of 414/100,000 [3]. Most maternal deaths occur during labor, delivery and the immediate postpartum period. Obstetric hemorrhage is the main direct cause accounting for 25% of maternal deaths, infections (15%), unsafe abortion (13%), eclampsia (12%), and obstructed labor (8%) [4]. Majority of maternal deaths in Kenya are due to obstetric complications that could have been prevented with adequate medical care during and after delivery [5]. The safe motherhood initiative launched in Nairobi (1987), aimed at raising awareness about the numbers of women dying each year from complications of pregnancy and childbirth, and to challenge amelioration of the situation [6]. There are clear strategies and specified interventions for the reduction of maternal morbidity and mortality, often referred to as the Pillars of Safe Motherhood. These include: safe delivery, antenatal care, postnatal care and family planning [7]. Safe delivery ensures that all deliveries are attended by persons with the right knowledge, skills and equipment and also provide post-partum care to mother and baby [8]. In an effort to reduce maternal mortality, the indicators of progress are Maternal Mortality Ratio (MMR) and proportion of births attended by skilled attendants [9,10].

Globally, one third of births take place at home without the assistance of a skilled attendant [11]. In Africa, less than 50% of births are attended by a skilled health worker [12] despite an increase from 43% to 57% between 1990 and 2005 in all developing regions. Consequently, two million women have died in Africa during childbirth since 2000 [9]. According to Kenya Demographic Health Survey (2008/9), the percentage of medically assisted deliveries has fallen consistently from 50% in 1993 to 44% of births in 2008; 28% of the mothers have a traditional birth attendant, 21% of mothers are assisted by relatives and/or friends and 7% deliver alone [3]. In Kenya, skilled birth attendants' definition is restricted to doctors, nurses and midwives. Traditional Birth Attendants are excluded from this definition as most of them (80%) lack formal training in pregnancy and birthing related matters [13]. Skilled attendants seem to be in larger numbers in urban areas where the numbers of health facilities are also more than in the rural areas. Kenya's government run health facilities are under-financed and characterized by shortages of most basic essentials including a dire shortage of personnel where only 15% of all Kenyan health workers providing maternal health services have received any type of in-service training in treating delivery-related complications and yet (83%) of expectant mothers access the facilities during delivery [3,14]. Payment for health care services in Kenya is both out of pocket where the clients pay cash and also third party system where clients who subscribe to insurance fund use their cover to pay for the medical services. Recently the government has directed that pregnant women should not pay delivery fees at any government run health facilities [15]. Efforts to reduce maternal mortality and morbidity must address societal and cultural factors that affect women's health and their access to services [14]. In Nyandarua South District, inadequate access to integrated, affordable and quality reproductive health (RH) services especially by youth and adolescents, unplanned pregnancies, little male participation in RH issues and unsafe motherhood have been reported to influence provision of reproductive health services [16]. This study aimed at establishing delivery practices among selected mothers seeking child welfare services at selected health facilities in Nyandarua South district, Kenya, to determine the proportions of deliveries attended by skilled birth attendants as well the factors influencing the mothers' choices.

## Methods

The study was carried out in Nyandarua South district, Kenya. The district is amongst districts with unsafe motherhood as an issue of concern in Kenya [16]. The district has an area of 1,367.2 square kilometers and is divided into 3 administrative divisions. Based on the National Population Census, the district has a total population of 230,622 with an annual growth rate of 3.3% [16]. The crude birth rate (CBR) is 39.2% and a total fertility rate (TFR) of 6.6. Population of special health significance include: infant population (10,861), children under five years is (48,357) and 11,655 women in the reproductive age group (15-49 years) [16].

This was a descriptive cross-sectional study where the study population comprised of mothers aged 15 to 49 years attending Maternal Child Health (MCH) clinics at the district and sub-district hospitals, and who had a live birth in the two years preceding the survey while in the study area. Delivery practice in this study was defined as the type of care a mother utilized during delivery with regard to the place of delivery and type of attendant during delivery. Using the estimated proportion of deliveries attended by skilled attendants (42%) in Kenya as reported by the most recent demographic data available at the time of the study [17], the sample size was calculated using the Fisher formula [18].

Using a sampling frame estimated from using the average number of mothers visiting the clinic per day (established from the facility), multiplied by the number of days to be spent on the site, a random sample of 416 mothers were selected to participate out of which 409 gave successful interviews. To calculate the sampling interval, the total estimated sampling size was divided by the calculated sample size giving an interval of three thus every third mother was recruited systematically. This was done every day until the desired sample size was realized. A structured questionnaire designed in English but administered by the researcher and/or trained research assistants in *Kikuyu *(local language) was used to collect data. Respondent mothers were asked about their demographic characteristics (age, education level, marital status, number of deliveries), socio-economic data (type of household and number of sources of income), practices and perceptions (place of delivery, birth attendants, antenatal attendance, spouse involvement in reproductive health issues, experiences during their last delivery). Satisfaction, practice and knowledge scores were generated by the researcher using different elements from the questionnaire each with 100 scores.

Data captured in questionnaires was entered into Access database and cleaned. Data analysis was performed using Statistical Package for Social Sciences (SPSS Vers. 12.0 inc., 444 N. Michigan Ave. Chicago Illinois). Analysis of safe and unsafe delivery practices among the 409 mothers was carried out using the most recent delivery report. Definition of safe or unsafe practice was based on the skills of the personnel that assisted in the delivery. Safe delivery was considered to be one that was attended by a skilled birth attendant. Analysis of first and last delivery reports excluded mothers who had delivered only once at the time of the study. Differences in proportions were compared using the Pearson's chi-square test for the categorical variables. A two-sided P-value < 0.05 was considered statistically significant. Binary logistic regression was used to eliminate confounding factors and assess the effect of various factors on place of delivery and type of attendant at delivery. The six predictive factors which significantly associated (independently) with type of delivery care in bivariate analysis were included in the model and their effects examined. These factors (independent variables) included: age of the mother, total number of deliveries in a life time, mothers level of education, perception on home versus hospital attendants, satisfaction and knowledge scores. The dependent variable was delivery practice which was dichotomized as delivery by skilled birth attendant coded as zero and delivery by unskilled birth attendant was coded as one. Variables with P < 0.05 in the logistic regression were considered to predict delivery practice. Approval to carry out the study was obtained from Kenya Medical Research Institute (KEMRI) Scientific/Steering and National Ethical Review Committees. Only those mothers, who met the study requirements, verbally consented and voluntarily signed the consent forms were enrolled into the study. Participants who could not who could not write indicated their consent by a fingerprint, which was witnessed by the interviewer.

## Results

### Maternal Characteristics

The demographic characteristics of the study population are as shown in Table 1. The total number of interviewed mothers was 409. The overall mean age of the mothers was 28.1 + 6.4 years ranging from 15 to 49 years with the majority (47.2%) aged 22 - 28 years. Majority of the mothers 71.1% (291) had acquired primary level education with reduced numbers with higher levels of education. The mean number of live births was 3. More than 50% of the mothers had delivered less than 3 children in their lifetime at the time of the study.

**Table 1:** Demographic characteristics and Distribution of deliveries by type of attendant and place of delivery among the respondent mothers who had a live birth in the two years preceding the survey in Nyandarua District, Kenya

Characteristics	N	%†
**Mean age [SD] years**	28.1 [6.4] ^◦^	
**Marital status**:		
Married	354	86.6
Single	43	10.5
Separated	11	2.7
Widow	1	0.2
**Level of education**:		
None	1	0.2
Primary	290	70.9
Secondary	100	24.4
College	16	3.9
University	2	0.5
**Religion**:		
Christian	385	94.1
Muslim	5	1.2
Traditional	1	0.2
No religious affiliation	18	4.4
**Number of children**:		
Mean number Alive [SD]	3 [2]^◦^	
Mean number Dead [SD]	1 [1]^◦^	
Mean number Total [SD]	3 [2]^◦^	
Less than 3 children	293	71.6
More than 3 children	116	28.2
**Wealth score**:		
<5	70	17.1
5-10	330	80.7
>10	9	2.2
**Delivery Attendant**:		
Doctor, nurse, midwife	564*	48.2
Neighbor and/or relative	452*	38.6
Self	137*	11.7
TBA	17*	1.5
**Place of Delivery**:		
Health Facility	562*	48
Home	608*	52

Abbreviations: SD, standard deviation; TBA, traditional birth attendant; * Number of deliveries; † Column percentages

### Delivery Attendance

Among the 409 mothers who participated in the study, a total of 1170 deliveries were reported during the study period (August-October) which included live and still births. This number of deliveries reported included prior delivery history of the mothers. Skilled birth attendants (doctors, nurses and midwives) attended 48.2% (564) of these deliveries while the rest were attended by unskilled birth attendants (neighbors, relatives and Traditional Birth Attendants (TBA)). Among the deliveries attended by skilled birth attendants, 2.7% (15) were at home while 97.3% (549) were in a health facility. In 51.8% (606) of the deliveries that were attended by unskilled birth attendants, 2.1% (13) were in a health facility where the mother either delivered alone or was attended by her companions. Majority of the mothers 72.3% (225) were attended by skilled birth attendants during their first delivery compared to 54.3% (169) who were attended by unskilled birth attendants during their most recent delivery. Consequently, 73% (227) of the mothers delivered in a health facility during their first delivery compared to 57.2% (178) during their most recent delivery. Over half (55.3%) of the mothers interviewed did not have a birth plan with 58.4% of them having their place of delivery being decided upon by their husbands.

There was a significant association between the first place a mother delivered and the last place (OR 3.9, 95% CI 2.3-6.6, P < 0.001) among the mothers who had delivered more than once. During her subsequent deliveries, a mother was 3.9 times more likely to deliver in the same place they delivered during their first delivery. Among the 409 participants, 51 (12.5%) had never delivered in a health facility. Among them, 27.5% (14) said that they were willing to deliver in a maternity facility in future while 72.5% (37) were not. The most frequently mentioned reasons for never delivering in a health facility were; perceived delays in being attended (56.9%), fear of episiotomy (25.5%) while 17.9% felt it was risky to deliver at home and were therefore willing to deliver in a health facility. Among the reasons given by the mothers for the choice of delivery place were; perceived comfort in the particular place (23.2%), distance to the health facility (18.3%), and type of antenatal care given in a health facility (16.6%). Based on all variables, 22.1% (79) of the respondent mothers were satisfied with health facility delivery services while 77.9% (279) were not.

### Factors associated with delivery practice among respondent mothers

#### Bivariate analysis

There was a significant association between mother's level of education and delivery practice (OR 55.3, 95% CI: 4.7-1539.9, P < 0.001). Majority 97.2% (139) of unskillfully attended deliveries were reported by mothers who delivered at home during their recent delivery (OR 581.5, 95% CI 174-2152.3, P < 0.001). Mothers who perceived unskilled birth attendants as similar 61.3% (68) to the trained health care workers, in attending to deliveries were approximately 4 times more likely to be attended to unskilled birth attendants compared to those who acknowledged there was a difference in delivery attendance (OR 3.9, 95% CI 2.4-6.3, P < 0.001). Younger mothers were more likely to utilize skilled attendants during delivery than their elder counterparts (p = 0.001). There was also a significant association between parity and the type of delivery attendance (P < 0.001). Satisfaction with maternity services was associated with more facility based deliveries (Table 2).

**Table 2:** Association of selected factors with delivery by unskilled birth attendant among mothers seeking child welfare services in selected health facilities, Nyandarua South, Central Kenya

Factor	n (%*) delivery by unskilled birth attendant (N = 154)	n (%*) delivery by skilled birth attendant (N = 255)	Bivariate analysis, OR (95% C.I.)
Mother's age			
>35	30 (53.6)	26 (46.4)	**2.5 **(1.0 - 6.1)
31 - 35	30 (47.6)	33 (52.4)	2.0 (0.8 - 4.7)
26 - 30	46 (36.8)	79 (63.2)	1.3 (0.6 - 2.8)
21 - 25	37 (28.5)	93 (71.5)	0.9 (0.4 - 1.9)
<= 20	11 (31.4)	24 (68.6)	Reference
Mother' level of education			
Primary (class 1-3)	13 (76.5)	4 (23.5)	**55.3 **(4.7-1539.9)
Primary (class 4-8)	125 (45.6)	149 (54.4)	**14.3 **(2.0-291.5)
Secondary	15 (15.0)	85 (85.0)	3.0 (0.4-64.9)
Tertiary	1 (5.6)	17 (94.4)	Reference
Place of delivery attendance			
Home	139 (97.2)	4 (2.8)	**581.5 **(174.9-2152.3)
Health facility	15 (5.6)	251 (94.4)	Reference
Total number of deliveries in a life time			
>3 deliveries	74 (63.8)	42 (36.2)	**4.7 **(2.9 - 7.6)
< = 3 deliveries	80 (27.3)	213 (72.7)	Reference
Perception on birth attendants at home versus at health facility			
Similar	68 (61.3)	43 (38.7)	**3.9 **(2.4-6.3)
Not similar	86 (28.9)	212 (71.1)	Reference
Scores of mothers' satisfaction with maternity services (%)			
0 - 20	97 (53.3)	85 (46.7)	**4.6 **(2.5-8.8)
21 - 40	17 (32.7)	35 (67.3)	2.0 (0.9-4.6)
41 - 60	5 (21.7)	18 (78.3)	1.1 (0.3-3.8)
61 - 80	17 (27.9)	44 (72.1)	1.6 (0.7-3.6)
81 - 100	18 (19.8)	73 (80.2)	Reference
Scores of mothers' knowledge on safe delivery (%)			
0 - 20	17 (94.4)	1 (5.6)	**75.1 **(9.7-1587.3)
21 - 40	38 (77.6)	11 (22.4)	**15.3 **(6.4-37.2)
41 - 60	32 (41.0)	46 (59.0)	**3.1 **(1.6-6.1)
61 - 80	43 (32.1)	91 (67.9)	**2.1 **(1.1-3.9)
81 - 100	24 (18.5)	106 (81.5)	Reference
Wealth score			
Low	69 (39.0)	108 (61.0)	1.1 (0.7 - 1.7)
High	85 (36.6)	147 (63.4)	Reference

Abbreviations: N, total number of respondents; CI, confidence interval; OR, odds ratio; AOR, adjusted odds ratio; *Column percentages; Significant odds ratio values (unadjusted) printed in bold

#### Multivariate analysis

Binary logistic regression using the backward conditional method was performed on multiple factors to eliminate confounding factors and examine the effect of the seven predictive factors which significantly associated (independently) with delivery practice at bivariate analysis. Four factors were found to predict delivery practice among the mothers (Table 3). Mothers who had unskilled birth attendance were more likely to have <3 years of education (AOR 19.2, 95% CI 1.7 - 212.8) and with more than three deliveries in a life time (AOR 3.8, 95% CI 2.3 - 6.4. Mothers with perceived similarity in delivery attendance among skilled and unskilled birth attendants were associated with unsafe delivery practice (AOR 1.9, 95% CI 1.1 - 3.4). Low knowledge on safe delivery was associated with unskilled birth attendance (AOR 36.5, 95% CI 4.3 - 309.3).

**Table 3:** Logistic regression model for variables associated with delivery by unskilled birth attendant among mothers seeking child welfare services in selected health facilities, Nyandarua South, Central Kenya

Variables	Multivariate analysis, AOR (95% C.I.)
Mother's level of education	
Primary (class 1-3)	**19.2 **(1.7 - 212.8)
Primary (class 4-8)	5.9 (0.7 - 47.2)
Secondary	1.8 (0.2 - 15.7)
Tertiary	Reference
Total number of deliveries in a life time	
>3 deliveries	**3.8 **(2.3 - 6.4)
< = 3 deliveries	Reference
Perception on birth attendants at home versus at health facility	
Similar	**1.9 **(1.1 - 3.4)
Not similar	Reference
Scores of mother's knowledge on safe delivery (%)	
0 - 20	**36.5 **(4.3 - 309.3)
21 - 40	**7.4 **(3.0 - 18.4)
41 - 60	**2.2 **(1.1 - 4.4)
61 - 80	1.7 (0.9 - 3.1)
81 - 100	Reference

Abbreviations: CI, confidence interval; AOR, adjusted odds ratio; *Column percentages; Significant odds ratio values (adjusted) printed in bold

Dependent variable: (0 = Safe (Skilled birth attendance, 1 = Unsafe (Unskilled birth attendance)

## Discussion

A measure of the proportion of deliveries assisted by skilled attendants is one of the indicators of progress towards Millennium Development Goal 5, which aims at improving maternal health [9]. Skilled attendants are important during pregnancy, child birth and the immediate postnatal period. Considering the most recent delivery a mother had, the findings of this study showed that utilization of skilled delivery care among the mothers interviewed was slightly above fifty percent (54.3%) and hence it was still low. Majority of safe deliveries took place in health facilities hence the need to encourage women to deliver in health facilities where they can be attended by skilled attendants as well as get emergency attention in the event of complications. The results of this study are similar to those of other developing countries, where attendance of deliveries by skilled health care workers is still low [13]. Although most countries have reported notable increase in the proportion of births attended by skilled birth attendants, the slowest change has been noted in sub Saharan Africa, where the proportion of deliveries attended by skilled birth attendants went up from 40 per cent in 1990 to 43 per cent in 2000 showing a progress rate of 0.1% which is far below the 5.1% required to achieve MDG 5 which aims at increasing the proportion of births attended by skilled birth attendants in that at least 90% of births worldwide be attended by skilled health personnel by 2015 [19]. In South East Asian and Northern African countries there has been increased coverage in births attended to by skilled health care workers from 55% in 1995 to 81% in the period 2000-2007 [20].

In Kenya, utilization of health facilities for labor and delivery services has been on the decline. According to Kenya Demographic Health Survey 2008, the percentage of medically assisted deliveries has fallen consistently from 50% in 1993 survey to 44% in 2008 [3]. Thus, based on all the deliveries a mother had prior to the study, the overall proportion of skilled attendance during delivery (48.2%), was slightly above the national estimate of births attended by skilled attendants (44%) as reported by the Kenya Demographic Health Survey [3]. From the findings of this study, it was surprising to find that a high proportion of the mothers gave birth without assistance (11.5%). This estimate was lower than the 18% observed in a study in Western Province [21] but almost twice the national average (8%). Other studies in Kenya have confirmed low skilled birth attendance of mothers during delivery with the situation warranting quick attention if MDG 5 targets have to be met [21,22]. Unskilled birth attendants are most likely to attend to deliveries in unhygienic conditions putting both the mother and the newborn at risk of delivery-related complications and this may lead to maternal and neonatal morbidity and mortality as lack of skilled attendance makes it difficult to seek assistance in the event of complications [23]. However, unlike other parts of the country such as Mwingi and Kwale Districts, where TBAs attend to over 70% of the deliveries, [23] only 1.5% of all the deliveries reported in this study were attended by TBAs. This estimate was also quite low compared to the nationally observed rate of 28% [3] attendance. This was probably due to the fact that the health facility based population of study would have been different from that in the community. However, TBAs are popular among communities that highly guard their cultural beliefs associated with child birth [24]. In a recent study carried out in the slums of Nairobi Kenya, TBAs suggested that hospital-based providers tend to disregard people's cultural beliefs, maintaining that in hospitals, little or no attempt is made to find out and respect the cultural beliefs and preferences of patients [24]. In this study, no cultural beliefs associated with pregnancy and child birth were identified and this could also explain the low proportion of TBA attended deliveries. In other studies, mothers are found to place high value on TBA services as they not only attend to deliveries but also give personalized care such as helping with household chores [25,26]. Kenyan TBAs with formal training have been accused of frequently sliding back into their old ways of managing birth, often with disastrous consequences for the women who use their services [27]. Though in this study it was not established whether the TBAs were trained or not, TBAs have been found to form the backbone of maternity services in rural areas and are also relied upon by large populations that have poor and/or no access to health facilities [28,29]. There is no clear policy in Kenya on whether TBAs should continue to attend to deliveries hence their activities are not regulated, although United Nation Population Fund Agency (UNFPA) supports their training on how to conduct clean and safe deliveries [30].

Most maternal deaths can be averted if deliveries are overseen by skilled attendants [10] with the right knowledge and skills. From the findings of this study, mothers who perceived birth attendants at home (unskilled) as similar to birth attendants in health facilities (skilled) in the way they attend to deliveries were attended by unskilled birth attendants mainly relatives during delivery. In communities where TBAs are perceived as equally skilled as the medically trained health professionals (doctor, nurse, and midwife) in attending to deliveries, they enjoy preference to attend to deliveries over the skilled attendants. The perception of mothers on birth attendants could be greatly influenced by the interpersonal relations with the attendants during labor and delivery. These findings are consistent with those of an earlier study in rural Kenya on household characteristics that influence mothers' choice of a delivery place [31]. In this study, there was a significant reduction in hospital delivery attendance during the last delivery (57.2%) compared to the first (73%). Further analysis showed that there was a significant association (P < 0.001) between the first and last places of delivery where a mother was found to be 3.9 times more likely to deliver her last baby in the same place she delivered during her first delivery. This shows that the first experience a mother had at the chosen place of delivery and with the chosen birth attendant was very important. Moreover, the health care providers have an opportunity to enhance a positive relationship between themselves and their clients which would in turn lead to a positive perception on health facility based deliveries as well as on medically trained delivery attendants. Mothers who identified unpleasant procedures and delays in hospitals as dislikes for not delivering in health facilities felt that the health facility was a harsh setting for delivery and expressed no interest in delivering in health facilities whereas those who perceived home as a risky delivery place chose to have health facility based deliveries. In another study at Kwale district, Kenya it was revealed that women perceived health facilities as a harsh setting for childbirth and they therefore chose to deliver their babies at the comfort of their home [25]. The findings of this study were consistent with those of a study in Bangladesh where different perceptions and interpretations of danger signs during pregnancy and delivery were found to be important factors that influenced mothers' health seeking behavior during pregnancy and delivery [32]. Unlike an earlier study in Tanzania where mothers expressed fear and dislike of caesarian section during delivery [33], in this study, mothers mostly disliked episiotomy. These findings show that majority of the mothers did not deliver at home because they were unaware of the risks involved but because of their anticipation of the quality of maternity services they would receive.

In Kenya, the utilization of maternal services differs from district to district and from community to community. This difference in utilization is strongly attributed to maternal characteristics and accessibility factors [34]. The findings of this study showed that younger women were more likely to utilize formal maternity services during labor and delivery unlike the older women. Similarly, an earlier study in Kenya also associated delivery practice with maternal age [35].

As reported in this study, birth preparedness is an important factor which can influence mothers' choice of delivery practice [36,37]. From the results, 55.3% of the mothers did not plan on the delivery place prior to the delivery with majority, (58.4%) of them being decided for by their husbands. The results of this study shows that decision makers or household heads have a role to play in determining the place of delivery as well as the birth attendant and this has been reported in other countries [8,38,39]. The socio-economic status of mothers has been found to influence mothers' choice of medically assisted deliveries [25,31,40]. In this study, there was no significant difference between the wealth score among mothers who delivered unsafely and those who delivered safely (p > 0.05). This showed that both groups of mothers; those with high wealth score and those with low, had an equal chance of choosing the type of delivery care and therefore the socio-economic status was not an issue in choice of delivery practice. In Kenya, it is unlikely to have a skilled birth attendant attend to deliveries at home. Consequently, 97% of unskilled attendances were reported by mothers who delivered at home during their most recent delivery. Mothers who chose to deliver at home were 582 times more likely to use unsafe delivery practice compared to those who delivered at a health facility. This finding has been reported in other studies [8,21,22]. Service delivery in various maternity services is an important factor that can predict the utilization of those services. Findings showed that 53.3% (97) of the mothers, who expressed dissatisfaction with service delivery in the various maternity facilities they had attended for delivery, had unsafe delivery practices at home. The findings of this study are consistent with other studies carried out in Bangladesh, Malawi and Kenya where poor quality of maternity care services was identified as one of the factors contributing to low utilization of maternity services by mothers [8,31,34,38].

The current study showed that increase in the number of deliveries was predictive of the delivery practice where mothers who had three children and above were found to practice unsafe delivery as compared to those who had delivered less than 3 children. Other studies have also confirmed significance of parity with utilization of modern maternity services where older, higher parity mothers tend to use a health facility lesser than younger, lower parity mothers [21,22]. As was the case in this study, several studies have also identified mothers' level of education as one of the factors that determines choice of delivery place as well as of birth attendants [8,21,35,41]. Mothers with low primary education (class 1-3) were found to have 19.2 [95% CI 1.7 - 212.8] probability of being attended by unskilled attendants compared to those with tertiary education. Education level can therefore be related to the level of exposure to the right information with regard to delivery. Knowledge of risks involved during delivery was also significantly associated with the delivery practice. Majority (94.4%) of the mothers who had no correct information on safe delivery were found to get information on delivery from elderly women who may have been misinformed and they practiced unsafe delivery compared to 18.5% that had the correct information. The odds of practicing unsafe delivery increased with decreased knowledge on safe delivery with those with least knowledge likely to deliver unsafely (AOR 36.5, 95% CI 4.3 - 309.3). Knowledge of risks involved in unskilled birth attendance was linked to the source of information whereby uninformed sources could be misleading and thus result in incorrect choices on safe delivery. These findings were in agreement with those of a study carried out in South Africa where lack of awareness of maternity waiting homes was one of the reasons for non utilization of obstetric services [40].

## Conclusions

The mother's level of education, total number of deliveries in a life time, mother's perception on birth attendants at home versus at health facility and mother's knowledge scores on safe delivery (%) were found to be predictive of delivery practice. Mothers' anticipation of the maternity services influences their use of these services. Improving maternal health not only requires raising awareness of complications associated with pregnancy and childbirth but most importantly providing access to high quality reproductive health services that include; antenatal, delivery, postpartum and family planning. It would also be important to address the reasons given by the mothers that cause dissatisfaction with the services offered. This survey can provide baseline data for this rural area against which efforts to improve services in the delivery care can be evaluated. This being a cross sectional descriptive study, no comparisons were made. During data collection the study assumed that the information being given was true and not biased. More research should also be carried out to explore more factors that hinder utilization of safe delivery services at the community level as well as the perspectives of the health care providers on how the services can be improved at the operational level in order to provide better guidelines for planners, administrators and policy makers.

## List of abbreviations

CBR: Crude Birth Rate; DFID: Department for International Development; FCI: Family Care International; FGD: Focus Group Discussion; HIV: Human Immunodeficiency Virus; HMIS: Health Management Information System; KEMRI: Kenya Medical Research Institute; KDHS: Kenya Demographic Health Survey; MCH: Maternal Child Health; MDGs: Millennium Development Goals; MDR: Maternal Death Review; MMR: Maternal Mortality Rates/Ratio; MNPI: Maternal & Neonatal Programme Efforts & Index; OR: Odds Ratio; PRB: Population Reference Bureau; SPSS: Statistical Package for Social Sciences; TBAs: Traditional Birth Attendants; TFR: Total Fertility Rate; UNFPA: United Nations Population Fund; WB: World Bank; WHO: World Health Organization.

## Competing interests

The authors declare that they have no competing interests.

## Authors' contributions

CW was involved in the design of the study, data collection, in analysis and interpretation of the data, and in drafting the paper. EM was involved in giving technical guidance in the design of the study and in the revision of the manuscript. ZP and GB were involved in the revision of the manuscript. MM was involved in statistical analysis and interpretation of data. Each author has given final approval of the version to be published.

## Pre-publication history

The pre-publication history for this paper can be accessed here:

http://www.biomedcentral.com/1471-2458/11/360/prepub

## References

[B1] World Health Organization, UNICEF, UNFPA/WB Maternal mortality in 2005: estimates developed by WHO, UNICEF, UNFPA and World bank 2005 Geneva, Switzerland

[B2] Department for International Development DFID'S Maternal Health Strategy Reducing maternal deaths: evidence and action. DFID Second Progress Report 200721215871

[B3] Kenya National Bureau of Statistics, Ministry of H, O. R. C. Macro Kenya Demographic and Health Survey 2008 report 2009 Calverton, Maryland, USA

[B4] Ronsmans C Graham W Maternal mortality: who, when, where, and why' Lancet 2006 368 1189 2000 10.1016/S0140-6736(06)69380-X 17011946

[B5] Ministry of Public Health and Sanitation Annual Health Sector Statistics 2008 http://www.publichealth.go.ke

[B6] Starrs A Safe motherhood: 20 years and counting Lancet 2006 368 1130 1133 10.1016/S0140-6736(06)69385-9 17011924

[B7] World Health Organization Reconciling maternal, newborn and child health with health system development. Chapter 7 in World Health Report 'Make every mother and child count' 2005 Geneva, Switzerland: WHO

[B8] Kabir M Safe delivery practices in rural Bangladesh and its associated factors: Evidence from Bangladesh demographic and health survey-2004 East African Journal of Public Health 2007 4 2 67 72 18085134

[B9] United Nations The Millennium Development Goals report: Statistical annex 2007 New York http://www.un.org/millenniumgoals

[B10] Safe Motherhood Newsletter Making pregnancy safer: a strategy for action 2002 29 SMN

[B11] World Health Organization Proportion of births attended by a skilled health worker 2008 Geneva, Switzerland: WHO http://www.who.int/reproductivehealth/publications/maternal_perinatal_health/2008_skilled_attendant.pdf 2008 updates

[B12] World Health Organization Skilled attendant at birth 2006 updates 2006 Geneva, Switzerland

[B13] Tawiah E Maternal health in five Sub-Saharan African countries. Poster presentation at The Fifth African Population Conference, 10-14 December 2007 2007 Arusha International Conference Center, Arusha

[B14] Kenya Service Provider and Assessment Survey KSPAS Maternal and Child health, Family planning and Sexually Transmitted Infections 2006 National Coordination Agency for population and Development, MOH, CBS, ORC, Macro USA: USAID, UNICEF, DFID

[B15] Wanja J Hospitals faulted for detaining women; "Don't pay maternity fees, urges minister' The Daily Nation 2010 8

[B16] Nyandarua South District Strategic Plan Implementation of the National Population Policy for Sustainable Development. National Coordination Agency for Population and Development 2008

[B17] Population Reference Bureau World Population Data Sheet 2005 Washington, DC: Population Reference Bureau http://www.prb.org/pdf05/05worlddatasheet_eng.pdf

[B18] Fishers A Andrew E Townsend W Hand book for family planning operations research designs 1998 2 USA: Population Council

[B19] United Nations Population Fund UNFPA Key actions for the further implementation of the Programme of Action of the International Conference on Population and Development. Adopted by the Twenty-first Special Session of the United Nations General Assembly 1999 New York: UNFPA 64

[B20] World Health Organization The Partnership for MNCH: Ten-Year Strategy. The World Bank 2007. Healthy Development. The World Bank Strategy for Health, Nutrition and Population Results. Concept paper Global Business Plan for MDG 4&5 2007 Geneva, Switzerland: WHO

[B21] van Eijk A Bles H Odhiambo F Ayisi J Blokland I Rosen D Adazu K Slutsker L Lindblade K Use of antenatal services and delivery care among women in rural Western Kenya: a community based survey Journal of reproductive health 2006 3 2 1 9 10.1186/1742-4755-3-2 PMC1459114 16597344

[B22] Mwaniki P Kabiru E Mbugua G Utilisation of antenatal and maternity services by mothers seeking child welfare services in Mbeere District, Eastern Province, Kenya East African Medical Journal 2002 79 184 187 12625672 10.4314/eamj.v79i4.8875

[B23] MOH/UNFPA Needs assessment of obstetric fistula in Kenya 2004 Final report by division of reproductive health http://www.endfistula.org/docs/na_kenya.pdf

[B24] Izugbara C Ezeh A Fotso JC The persistence and challenges of homebirths: perspectives of traditional birth attendants in urban Kenya Health Policy and Planning 2009 24 36 45 19033324 10.1093/heapol/czn042

[B25] Cotter K Hawken M Temmerman H Low Use of Skilled Attendants' Delivery Services in Rural Kenya Journal of health, population and nutrition 2006 24 4 467 471 PMC3001150 17591343

[B26] Mutambwira J Pregnancy, Childbirth, mother and child care among the indigenous people of Zimbambwe International Journal of Gynaecology & Obstetrics 1985 23 275 285 10.1016/0020-7292(85)90021-9 2866114

[B27] Kamal IT The traditional birth attendant: a reality and a challenge International Journal of Gynecology & Obstetrics 1998 63 Suppl. 1 S43 S52 10075211 10.1016/s0020-7292(98)00183-0

[B28] Smith J Coleman N Fortney J Johnson J Blumhagen D Grey T The impact of traditional birth attendant training on delivery complications in Ghana Health Policy Plan 2000 15 326 331 10.1093/heapol/15.3.326 11012408

[B29] Bisika T The Effectiveness of the TBA Programme in Reducing Maternal Mortality and Morbidity in Malawi East African Journal of Public Health 2008 5 2 103 110 19024419

[B30] United Nations Populations Fund Support to Traditional Birth Attendants Evaluation Findings 1996 7 UNFPA

[B31] Hodgkin D Household characteristics affecting where mothers deliver in rural Kenya Health Economics 1996 333 340 10.1002/(SICI)1099-1050(199607)5:4<333::AID-HEC202>3.0.CO;2-K 8880170

[B32] Caldwell BK (Ed) Identification of unmet needs. Why women continue to die from childbirth in Dhaka, Bangladesh Interregional seminar on reproductive health, unmet needs and poverty: Issues of access and quality of health 2002 Bangkok

[B33] Mwifadhi M Brigit O Joanna A Rachel A Haws A Hassan M Marcel T David S The use of antenatal and postnatal care: perspectives and experiences of women and health care providers in rural southern Tanzania BMC Pregnancy and Childbirth 2009 9 10 http://www.biomedcentral.com/1471-2393/9/10 19261181 10.1186/1471-2393-9-10 PMC2664785

[B34] Jane T Karen H Robert J Marcia A Access, quality of care and medical barriers in family planning programmes International Family Planning Perspective 1995 21 64 69 10.2307/2133525

[B35] Magadi M Madise N Diamond I Factors associated with unfavourable birth outcomes in Kenya Journal of Biosocial Science 2001 33 199 225 10.1017/S0021932001001997 11284627

[B36] Mulongo E Witte K Bajunire F Nabukera S Muchunguzi V Batwala F Farr C Barry S Birth plan and health facility based delivery in rural Uganda East African Medical Journal 2006 83 74 83 16771103 10.4314/eamj.v83i3.9401

[B37] Mutiso SM Qureshi Z Kinuthia J Birth preparedness among ANC clients East African Medical Journal 2008 85 6 474 478 10.4314/eamj.v85i6.9625 18817024

[B38] Seljeskog L Sundby J Chimango J Factors Influencing Women's Choice of Place of Delivery in Rural Malawi-An explorative study African Journal of Reproductive Health 2006 10 3 66 75 10.2307/30032472 17518132

[B39] Mswia R Lewanga M Moshiro C Whiting D Wolfson L Hemed Y Alberti K Kitange H Setel P Community based monitoring of safe motherhood in the United Republic of Tanzania Bulletin of the World Health Organization 2003 81 2 87 94 12751416 PMC2572394

[B40] Uyirwoth G Itsweng M Mpai S Nchabeleng E Nkoane H Obstetrics service utilization by the community in Lebowa, Northern Transvaal East African Medical Journal 1996 73 2 21 27 8756046

[B41] Nwakoby BN Use of obstetric services in rural Nigeria Journal of Reproductive and Social Health 1994 114 132 136 10.1177/146642409411400304 7932482

